# Adopting and Adapting a Falls Prevention Program: Lessons Learned From Implementing a Model From a Different Context

**DOI:** 10.1093/geroni/igab046.1423

**Published:** 2021-12-17

**Authors:** Alexandra Morelli, Carol Petrie, Christine Ferrone, Phillip Clark

**Affiliations:** University of Rhode Island, Kingston, Rhode Island, United States

## Abstract

Geriatric Workforce Enhancement Programs (GWEPs) are ideally suited to develop and implement educational programs to transform the geriatric care system. They link academic programs, clinical partners, and community-based organizations to bridge care system gaps to improve the health and social care of older adults. Such a collaboration is especially important in falls prevention, where primary care assessments generate referrals to community programs that enroll older adults to reduce their risk of falling. However, exporting an evidence-based model developed in one context for implementation in another is not without its perils and pitfalls. This paper explores the challenges of applying a model developed elsewhere to the Rhode Island context, including the need to understand how structural differences in academic, primary care, and community-based systems require flexibility, innovation, and persistence in overcoming the networking challenges in these different settings. Recommendations for implementing program models in a variety of settings are explored.

